# Reported Resignation, by Prof. C. A. Lee, of the Chair of Materia Medica, in the Buffalo Medical College

**Published:** 1867-12

**Authors:** 


### Reported Resignation, by Prof. C. A. Lee, of the Chair of Materia Medica, in the Buffalo Medical College.

On account of ill-health, during a portion of last summer, Prof. Lee offered resignation of his professorship, which, however, was not accepted by the Faculty, and returning health has enabled him to discharge its duties with his usual vigor and ability. We are happy to be able also, to state, that his aims and purposes, in this respect, have changed, and that he no longer desires the acceptance of his offered resignation.

---

The Physicians' Visiting List, for 1868. Philadelphia: Lindsay & Blackiston.

Messrs. Lindsay & Blackiston have again published their popular Physicians' Visiting List, for 1868. This little work has enjoyed a most deserved reputation amongst physicians for the completeness and compactness of its arrangement, and its success demonstrates its value. Its table of contents is as follows: Almanac; Table of Signs; Marshall Hall's ready Method in Asphyxsia; Poisons, and their Antedotes; Table for caculating Utero-gestation; Blank leaves for visiting list, monthly memoranda, address of patients, nurses, accounts, memoranda of wants, obstetric and vaccination engagements, record of births and deaths, general memoranda, etc. This visiting list appears to us as superior to most books of similar kind, and certainly these pocket records now appear to be regarded as indispensible.

